# Women's perceptions of antenatal, delivery, and postpartum services in rural Tanzania

**DOI:** 10.3402/gha.v8.28567

**Published:** 2015-10-22

**Authors:** Gladys Reuben Mahiti, Dickson Ally Mkoka, Angwara Dennis Kiwara, Columba Kokusiima Mbekenga, Anna-Karin Hurtig, Isabel Goicolea

**Affiliations:** 1 Department of Development Studies, School of Public Health and Social Sciences, Muhimbili University of Health and Allied Sciences, Dar es Salaam, Tanzania; 2 School of Nursing, Muhimbili University of Health and Allied Sciences, Dar es Salaam, Tanzania; 3 Division of Epidemiology and Global Health, Umea University, Umea, Sweden

**Keywords:** health facility, maternal health, women's perceptions, postpartum, Tanzania

## Abstract

**Background:**

Maternal health care provision remains a major challenge in developing countries. There is agreement that the provision of quality clinical services is essential if high rates of maternal death are to be reduced. However, despite efforts to improve access to these services, a high number of women in Tanzania do not access them. The aim of this study is to explore women's views about the maternal health services (pregnancy, delivery, and postpartum period) that they received at health facilities in order to identify gaps in service provision that may lead to low-quality maternal care and increased risks associated with maternal morbidity and mortality in rural Tanzania.

**Design:**

We gathered qualitative data from 15 focus group discussions with women attending a health facility after child birth and transcribed it verbatim. Qualitative content analysis was used for analysis.

**Results:**

‘Three categories emerged that reflected women's perceptions of maternal health care services: “mothers perceive that maternal health services are beneficial,” “barriers to accessing maternal health services” such as availability and use of traditional birth attendants (TBAs) and the long distances between some villages, and “ambivalence regarding the quality of maternal health services” reflecting that women had both positive and negative perceptions in relation to quality of health care services offered’.

**Conclusions:**

Mothers perceived that maternal health care services are beneficial during pregnancy and delivery, but their awareness of postpartum complications and the role of medical services during that stage were poor. The study revealed an ambivalence regarding the perceived quality of health care services offered, partly due to shortages of material resources. Barriers to accessing maternal health care services, such as the cost of transport and the use of TBAs, were also shown. These findings call for improvement on the services provided. Improvements should address, accessibility of services, professionals' attitudes and stronger promotion of the importance of postpartum check-ups, both among health care professionals and women.

The provision of appropriate maternal health care remains one of the main challenges in developing countries (1–4). There is agreement that the provision of quality clinical services is essential if high rates of maternal death are to be reduced (5). Nevertheless, a focus on tackling the clinical causes of maternal death may not be the most comprehensive perspective from which to understand the problem of maternal mortality, since it does not highlight the relevance of the social determinants of health (6, 7). It is important to consider other factors that influence the risk of dying during pregnancy, delivery, and postpartum. Such factors include socio-economic status, education, and ethnicity. Acknowledging the importance of social determinants in understanding the strongly unequal distribution of maternal deaths – which is highest among the poorest people in sub-Saharan Africa (8) – is therefore needed (3). From that perspective, neglect of maternal health constitutes not merely a public health issue, but also a violation of women's right to the highest attainable standard of health care (9–11).

From a rights-based approach, states are responsible for: 1) reducing inequalities that limit the opportunities of certain groups of women to enjoy safe motherhood and 2) providing an adequate array of maternal health care services that are available, accessible, acceptable, and of good quality (11–13). Professionals and researchers could assess these four criteria in different ways. However, from a rights-based approach, the ultimate and most relevant assessment should be provided by the users of such an array of services, connected to the principles of accountability and participation. To this end, it becomes especially relevant to explore the perceptions of the women towards whom this array of maternal health care services is directed.

## The right to maternal health services in Tanzania

Tanzania has a strong policy towards tackling maternal mortality and morbidity. In the 2007 Tanzanian health policy, maternal and child health were considered the ‘prime targets for health care delivery’ (14). In subsequent years, a number of plans and programmes were implemented. The National Road Map Strategic Plan to Accelerate Reduction of Maternal, Newborn and Child Deaths 2008–2015 reflects the current global approach to maternal mortality reduction based on the continuum of care (11). These efforts have contributed to the decline from 578 maternal deaths per 100,000 live births in 2005 to 454 in 2010 (15). However, the challenge to reach the targeted maternal mortality ratio of 133 in 2015 is enormous. Utilisation of maternal health care services in health facilities is high during the antenatal period with at least one visit (96%), but decreases sharply when it comes to delivery (50%) and first postpartum follow-up visit (35%) (15).

Both the distribution and utilisation of health care facilities remain unequal (9, 10). Despite the focus of health policy on rural areas (where 80% of the Tanzanian population lives) (2), urban areas remain far better covered by health services and their utilisation is greater (15, 16). Large inequalities exist in institutional delivery and postpartum coverage between women depending on their education, socio-economic status, and the area where they live (see Table 1).

**Table 1:** Distribution of maternal health services in Tanzania

Distribution by region, education, and wealth status	Antenatal coverage (at least one visit) as compared to the four visits needed (%)	Skilled delivery coverage (delivery at health care facility) (%)	Postpartum service coverage (%)
Geographical area			
Rural	95.1	41.9	30.5
Urban	98.6	82.4	51.5
Education			
No education	93.5	33.8	23
Secondary +	97.8	84.6	58.5
Wealth			
Lowest quintile	94.1	33.1	23.5
Highest quintile	98.9	89.6	55.7

Source: Ref. 16.

Previous research in Tanzania shows that: 1) the decision regarding where to seek care during delivery is not in the hands of pregnant women alone, but depends strongly on advice from relatives, traditional birth attendants (TBAs), and health professionals (17, 18); 2) the quality of health care service was perceived as deficient by users, thus discouraging many women from going to health facilities for delivery (17); and 3) interactions between health care providers and users, the distance of the facility and associated costs of travel, the advice of health workers on the place of delivery, and knowledge of danger signs, were associated with type of delivery and use of services (19).

Previous research on maternal health in Tanzania has favoured the perspectives of service providers. Published studies exploring women's perspectives on maternal health care services remain scarce (20–22). However, women's perceptions of the services available are especially relevant to informing programme implementation. The aim of this study was to carry out an exploration of postpartum women's views about the maternal health services (pregnancy, delivery, and postpartum period) they received at health facilities in order to identify where gaps in service provision may lead to lower quality of service and increased risks associated with maternal morbidity and mortality in rural Tanzania.

## Methods

### Setting

The study was conducted in Kongwa district, one of the five districts in the Dodoma region. The district has a population of 248,656 (23), 90% living in rural parts of the district. It is an agricultural district where people mainly engage in cultivation, livestock keeping, and trade (23). The region was purposely selected because of its rural characteristics. The study is part of a larger ongoing research project on health systems in the Dodoma region. The area has a poor transport network which presents difficulties for women from rural and remote parts of the district seeking health care services.

There are in total 46 health facilities in Kongwa district: one district hospital, four health centres, and 41 dispensaries. At the dispensary level, they offer Antenatal Care services where immunisations and PMTCT services are included, and they assist normal deliveries; when they are faced with obstetric complications, they refer the mother to the higher level. They also offer postnatal check-ups that include contraceptive provision and detection of complications. At the health centre level, they offer basic emergency obstetric care services. At the district hospital, they offer comprehensive emergency obstetric care (EOC). Nearby the district hospital, there is also a maternity waiting home (Chigonela). In the Dodoma region, 97.8% of pregnant women receive at least one antenatal check-up. The WHO recommends four antenatal care visits (24). There are no disaggregated data by regions showing women utilisation of ANC services per number of visits. At the national level, 3.8% of rural women receive only one visit, 54.5% receive 2–3 visits, and 39.5% receive 4 or more visits. Institutional delivery is estimated to be 45.9%, and only 33.8% of mothers receive postnatal check-ups (15).

A number of interventions were reported to be implemented in the district to improve maternal health, such as 1) training service providers in EMOC; 2) introducing a waiting maternity home (*Chigonela*) to the district hospital, where women who live far away or have complicated pregnancies can stay until delivery; 3) equipping facilities with help from non-governmental organisations (NGOs); 4) motivating TBAs by paying them to bring mothers to give birth at the facilities (currently stopped due to unavailability of funds from the government); and 5) implementing community sensitisation to delivery in health facilities.

A study conducted in Kongwa by Mahiti et al. (25) revealed that women were attended after child birth by TBAs who performed certain rituals (bathing, cooking, etc.) that were appreciated by the women. In that study, TBAs also reported that they lacked training in postpartum care and the links between them and formal health care facilities were perceived to be poor (25).

### Participants

Fifteen focus group discussions (FGDs) were conducted between April and August 2012, involving a total of 105 women after child birth, at Kongwa District Hospital and Ugogoni Health Centre. These facilities were purposively selected to include both a larger hospital and a smaller health centre. Women were recruited when they came to vaccinate their children; since vaccination coverage is quite high in the area we expected that women coming to vaccinate their children encompass a diversity in terms of educational level, age, and other characteristics. The participants were aged between 14 and 45 years. The majority of women had no formal education (43%) and most were engaged in farming activities (91%).

### Focus group discussions

FGD guides were prepared in English and later translated into Kiswahili, the mother tongue of the participants and the moderator of the FGD. The guide was composed of broad themes including the participants’ perceptions of the health facility services offered for pregnancy, child birth, and postpartum care; their experiences of consultations at health facilities during pregnancy, delivery, and postpartum; and their interactions with health care providers. Five FGDs were conducted in the under-five monitoring rooms and the remaining nine groups were conducted outside the health facility, under the trees. Participants themselves chose the place for the discussions.

Verbal consent was obtained from all the women before starting the FGDs; all the women approached were informed of the aim of the project, and the researcher read the consent form to them and they were informed that their participation was voluntary. The researcher asked the participants not to disclose any personal issues that they were not comfortable sharing with the group; also the participants were not to disclose issues that were discussed outside the group. Participants were free to withdraw from the study at any point, and could refuse to answer any question they felt uncomfortable sharing with the group. Their consent to participation in the FGDs was recorded and was part of the transcripts. At the start of each FGD, the participants also agreed on a suitable place to conduct the FGDs.

The FGDs lasted between 50 and 60 min and were audio-recorded. The first author moderated the discussion, and an assistant took notes and managed the recording equipment. The groups’ size ranged from 6 to 10 women. After the 15th group, it was felt that saturation point had been reached – that is, no new information was emerging to answer our research questions (26). Based on continuous reflections and preliminary analysis shared within the research team, we finished data collection at that point.

### Non-participant observation

Non-participant observation was also used, where the researcher observed waiting times, postpartum mothers’ interactions with service providers, and the environment at the health facilities: amenities, cleanliness, and bedding. The observation took 30–60 min, and it was conducted before or after the FDGs. The researcher did not observe clinical procedures, that is, observing women giving birth, check-ups at antenatal clinics, and postpartum check-ups, to respect the privacy of the mothers in the health workers’ rooms. The observations were recorded in a field book for proper analysis in order to triangulate the information obtained from the FGDs.

### Data analysis

All data from the FGDs on the women's perceptions, after child birth, of the maternal health services offered to them were transcribed verbatim and later translated into English to facilitate analysis by the research group. We did not consider segregation of participants based on characteristics such as educated and non-educated, or employment, since the majority of women in this setting have incomplete primary education and work at home and in subsistence agriculture; therefore, it would have been logistically complicated to identify and enroll a sufficient number of women with secondary or high education or working in other occupations. Qualitative content analysis was used to analyse the data, as described by Graneheim and Lundman (27). The FGDs were read several times to obtain a sense of the whole. The texts containing information on antenatal, delivery, and postpartum care were selected into ‘meaning units’. A process of condensation then identified condensed meaning units from the original meaning units. The condensed meaning units were then abstracted and labelled with codes using the Open Code software programme (28). The codes were later grouped into categories reflecting the manifest content of the text (see Table 2).

**Table 2:** Example of process of analysis

Meaning unit	Condensed meaning unit	Code^a^
There are plenty of them (TBAs). Women go to give birth. When you go to the village, there are so many women not going to the hospital. They deliver at home where the TBAs help them.	Plenty of TBAs and women in villages give birth with their help. Women do not go to the hospital.	TBAs assisting women to deliver.

a The code was later part of the category ‘Barriers to accessing maternal health services’.

### Methodological considerations

Several criteria are used in evaluating trustworthiness: credibility, transferability, dependability, and confirmability (27, 29). In this study credibility – that is, how well the data addressed the intended focus – was covered in several ways. The use of purposive sampling enabled the selection of participants who fulfilled the criteria for participation. Confidentiality was encouraged and the participants agreed not to share the discussion outside the group. This increased the credibility of the information produced. The involvement of more than one researcher in the process enhanced dependability by ensuring that the interpretations emerged in data through researchers’ triangulation. In addition, the first author, a native speaker with expertise in rural development, collected data. The description of the study context, selection criteria, and data collection and analysis process was complemented with quotes to allow readers to assess the transferability of the findings.

All the FGDs followed an FGD guide and allowed openness to new insights through open-ended questions. New issues that emerged were considered in subsequent data collection and the analysis process followed an emergent design that enhanced dependability. The translation of FGD guides from English to Kiswahili was intended to increase the free expression of participants, as the participants were more conversant in Kiswahili than in English. Before administering the instruments, there was back-translation from Kiswahili to English in order to check the accuracy of translation and to meet the criterion of confirmability.

### Ethical approval

The necessary ethical approval for conducting this study was granted from the Senate Research and Publication Committee, Muhimbili University of Health and Allied Sciences. Further, permission to conduct the study was obtained from the Kongwa district executive director and district medical officer.

Informed consent was obtained from each potential participant. The discussions were anonymous because despite being reproduced in the paper the identities of the participants are protected.

## Results

The participants were purposely selected with help from health care providers, who were informed about the general aim of the study. The women were recruited when they came to the hospitals for their babies’ vaccinations. Participants were divided by parity and there were 43 primipara (first-time mothers) and 62 multipara (mothers with more than one delivery).

From analysis of the data, three categories emerged that reflected the women's perceptions of maternal health care services: ‘mothers perceive that maternal health services are beneficial’; ‘barriers to accessing maternal health services’ such as availability and use of TBAs and the long distances between some villages; and ‘ambivalence regarding the quality of maternal health services’ referring that women had both positive and negative perceptions in relation to quality of health care services offered’.

### Mothers perceive that maternal health services are beneficial

#### Benefits in pregnancy and delivery

Women mentioned the advantages of maternal services during pregnancy and delivery. Services were used both at times of physical illness and during routine visits. Women recognised the advantages of these facilities as they provide investigations, for example, using ultrasound to see the development of the foetus in the womb, or treat diseases in pregnant women, such as malaria or HIV infection.

While pregnant a woman can encounter several problems, this is why they (nurses) have instructed us to come for monthly check ups for some incurable diseases like HIV … They will counsel on what pregnant women should do to avoid infecting the unborn babies. Similarly they would also give us medications such as sulphurdoxine or pyrimethamine to control malaria. (FGD 2, primiparous women)

Women also expressed concern that delivery problems cannot be solved by people who lack relevant knowledge and skills, such as people in the places from which they come. They believed that, within medical settings, staff were better prepared to deal with those problems.

Firstly, this (the health centre) is where there is more safety, you are assured of safe delivery, and in case of any complications then you are assured to be helped rather than delivering in the street (at home). There are good services for pregnant women delivering in hospital. (FGD 13, multiparous women)

Observations showed that health workers provide reproductive and nutrition education to the women at antenatal care clinics. Health workers begin by educating women before offering services. Women also recognised that they receive advice from health workers on how to use routine services and on danger signs. They have been advised by health workers on the importance of delivering at health facilities.

When pregnant a woman might have headache, swollen legs and bleeding. We have been instructed to come to the facilities immediately when experiencing these problems. (FGD 10, primiparous women)

#### Benefits during postpartum stage

The women were clear on the advantages of medical services during pregnancy and delivery, but this was less obvious in relation to the postpartum period. Women were confused as to the benefits of postpartum care services compared with the pregnancy and delivery stages. FGDs show that after delivery the main reason for women returning to the health facility was to check the baby. Most women did not feel that they should attend postpartum check-ups for themselves, unless they suffered from a stomach ache, dehydration or malaria, or required a check-up after a caesarean section. However, some women mentioned the importance of coming back at the health facilities during postpartum since there might be health problems emerging during postpartum.

In most cases after delivery mothers will visit the reproductive health section for the sake of their babies … unless you have a personal problem. But from what I know they often do because of the babies. (FGD 9, multiparous women)

You might find a woman who has given birth got a caesarean section, she might face complications such as malaria and after that she can visit health facilities for checkups. […] There are many problems so after giving birth there is a need to come back for check up of the mother and the baby as when you stay at home after two to three days you might die. (FGD 4, primiparous women)

Observations also showed that postpartum women were interested to see their babies’ progress in the under-fives clinics, where they can take their babies for vaccinations and weight monitoring. There were no check-ups at the health facility for postpartum women. They returned home after visiting the under-fives section.

### Barriers to accessing maternal health care services

The women mentioned external factors that impair their access to services, such as the availability and use of TBAs, and the long distances between some villages and medical facilities.

When asked about home deliveries, the participants described the practice of home deliveries assisted by TBAs. This was also indicated as a challenge in the reproductive health report for Kongwa district namely that women are still assisted by TBAs in the delivery of their babies. Additionally, women described how traditions, customs, and distance were among the factors influencing them to give birth at home.

They don't see any importance as they have historical beliefs from their parents that, whether they deliver at home it is fine […] that, it is not important to deliver at the health care facilities as at home there are grandparents (Traditional Birth Attendants) who have been assisting delivery and you will deliver. Eeeh! (FGD 3, multiparous women).

Though women reported being helped by these informal health providers within the community, they also believed that home delivery could lead to complications.

Some women do not come, they give birth at home. Other TBAs are not trained, they use herbal medicine to assist deliveries … and this leads to difficulties. (FGD 7, multiparous women)

Women reported that long distance travel was also a problem for them in seeking health services.

There is a village which is a bit far, in that village there is no dispensary nearby nor anything eeeh. Women deliver at home at the moment. (FGD 4, multiparous women)

When arriving at the dispensary the woman pays them 30,000/= Tshs (14.16 USD). If she has to be referred to the hospital, she also has to pay them 10,000/= Tshs (4.72 USD) as a fine for delay. (FGD 3, multiparous women).

### Ambivalence regarding the quality of maternal health services

Women expressed conflicting views on the quality of the services offered. There were both positive and negative opinions regarding human and material resources.

#### Interactions with providers

Participants perceived that there was a shortage of human resources at the health facilities. Observations also indicated a shortage of human resources where women find nurses being multitasked, thus creating frustration for women who were eager to return home after child birth and carry out their household chores.

The health workers are not adequate. I am not sure if the numbers have increased recently. You might find that you have one nurse stamping the registration form, vaccinating children, weighing the under-five babies and dispensing drugs. The same person can have four to five responsibilities at a time. (FGD 10, primiparous women)

Some women recognised and appreciated the commitment of staff and dedication to their work.

When women are pregnant up to delivery stage, they receive good services. For example, in my case, when in labour, health workers were so close to me. I had a big problem with blood pressure, I once fainted and the nurse was close to me until last minute of delivery … in fact our nurses are loving and comforting. (FGD 7, multiparous women)

However, even if women recognised the commitment of staff, they were also very critical regarding the way they were treated. They expressed how staff did not help them, for example, during delivery. Women described being left alone during delivery, how health workers responded with negative gestures and angry facial expressions towards patients in need of help. As one participant pointed out, ‘*Health workers look at the patients with a bad eye*’ (FGD 7, multiparous women):

Health workers use rude language … you might be at a final delivery stage and the baby is almost coming, when you call them, they are not there to help. When you begin pushing the baby, the nurse would tell you that the baby is yet to come out. For instance, last year my sister-in-law was delivering, and the nurse came when the (baby) was completely out. It is dangerous. (FGD 7, multiparous women)

Participants also revealed that some health workers were drunk on duty. Women questioned their professional practices, which are supposed to be performed with care.

Alcoholism exists. This problem happened to me: they mixed up my laboratory investigation results and filed them in another patient's file. He gave me results that were not requested to be investigated. (FGD 4, multiparous women)

#### Waiting times and informal payments

Long waiting times during consultation hours were both observed and reported as a problem in this setting. Participants described that the behaviour of health workers often delayed services unnecessarily and sometimes resulted in patients waiting for their fellow health workers to start services. Most women said that they might arrive early at the facility to receive services so that they could return home to perform their household chores, but the services would only be offered later. Women also remarked that there are instances when health care providers were busy discussing their own issues during official hours, neglecting patients waiting for the services. Observations also revealed delays before health workers began serving patients.

Services are not good, as you might arrive early in the morning, say at nine o'clock, and receive services very late. For example, you can find a doctor arriving at work station at nine or ten in the morning, and upon arriving at his desk he might skip people who arrived early in the morning and call patients who came late to attend them. (FGD 10, primiparous women)

Instances of informal payments like bribes among health workers were also reported. Women commented that some health workers harassed the patients or their relatives at the facilities, asking for petty bribes, for instance, money to buy soft drinks. There were reports of poor service if the family failed to give a certain amount of money to the health worker. However, women in this study also mentioned that, not all health workers demanded money.

I saw it happening to a certain woman when she arrived for delivery. Health workers asked for 5000/=Tshs (2.36 USD) for a soda (soft drink). But I must confess this has never happened to me. (FGD9, multiparous women)

#### Material constraints: drug shortages and dirtiness

Women noted that some free drugs were given during pregnancy as well as at the regional hospital in the case of complications. However, they also perceived a general shortage of material resources, namely medication, and a dirty environment. Women perceived that the drugs in facilities are inadequate, and they were occasionally advised by health workers to buy drugs from their medical stores.

It is hard for health services in terms of drug administration and we have been informed that there are no drugs for injection. It worsens the condition of the patient. (FGD 3, multiparous women)

When I came back I get drugs free and there is one of my neighbors, she was treated for two months. My baby also have been sick and received medication. (FGD 4, multiparous women)

Observations by the researcher showed that the district hospital environment was not clean. Sometimes, staff only began cleaning when it was made known that visitors in authority were coming. The bedding and toilets in the labour ward and surrounding area were dirty.

To be honest, the environment in the labour ward is dirty and untidy. Sometimes you may be forced to sleep on blood-stained bed sheets used by another woman. (FGD 8, primiparous women)

Due to these bad experiences, women stated that they have lost trust in the services they receive. This was indicated by the sometimes fatalistic attitudes of the women.

When you have health problems you run here (to the health facility). If you are lucky enough, they help you and you get the services. You thank God. (FGD 3, multiparous women)

## Discussion

This study has explored the views of women on antenatal, delivery, and postpartum care services in a rural district of Tanzania. The women perceived maternal health care services to be beneficial and showed great awareness of the benefits of antenatal check-ups and delivery in a health facility. They seemed to possess a high level of knowledge on the problems that may arise during pregnancy and delivery. They also considered that health facilities were better equipped to handle them. These findings correspond with the findings of other studies in Tanzania (22, 30). Participants’ perceptions of the benefits of health facility care during pregnancy and delivery did not apply to the postpartum stage. This is not striking, since studies from Tanzania and other sub-Saharan countries show that postpartum care has received scant attention (15, 31). However, despite the perceived general benefits of maternal health services, the uptake of such services is hindered by multiple barriers related to accessibility, acceptability, and low quality of available services.

Availability, accessibility, acceptability, and good quality are criteria that health services should fulfil in order to contribute to users’ right to health care (11, 32–34). Accessibility is dependent not only on the availability of services but also on geographical distances, alongside other barriers such as cost or gender issues. The present study indicates that transport problems influence delays in seeking care. Other studies have pointed out that distance and geographical inaccessibility contribute to many maternal deaths (7, 19, 35–38). Our findings align with the results from a survey conducted by Tanzania Demographic and Health Survey which showed that the main perceived barriers to women accessing health services were lack of money and long distances to health services (15).

Transportation means cost and our study even identified the existence of extra costs linked to unofficial (illegal) payments. As regards barriers associated with informal payments, other studies in Tanzania have also pointed out how the presence of informal (and illegal) user fees –that is, for services within the antenatal clinic – contributes to women's inability to access medical services for maternal health care (39). Another study carried out in rural Tanzania supports the argument that high direct payments, together with the costs of unofficial payments, are acute barriers to the use of maternal services (40).

In our findings, the use of TBAs was common even if policies to incentivise TBAs to make referrals to health facilities have been implemented in the district. It remains unclear whether the use of TBAs hinders access to health facilities or whether they become a resource due to women's lack of trust in existing health facilities and other barriers to access. Women might opt to use a TBA at home instead of giving birth in a health facility due to the distance to the health facility, and the unreliability of transport in case of emergency (26, 41, 42).

Acceptability deals with cultural issues such as the language used and the way services and health providers approach the people they are supposed to serve. Good quality refers to both technical competence and the fulfilment of users’ criteria; the way the providers interact with them, waiting times, arrangements to ensure privacy, and even the availability of female providers to perform certain gynaecological procedures (11, 32–34).

In this study, the patronising attitudes of health workers and poor quality of care were major problems revealed by the women users of these services. Women found themselves mistreated, left alone when in the labour ward and verbally abused. The perception that good quality care was only available for those able to pay bribes further diminished women's trust in the health care services offered. These findings are in line with other studies in rural Tanzania, South Africa, and Australia (18, 20, 23, 43). Unpleasant experiences with services discourage women from seeking them again, discourage other potential users, and possibly affect the degree of compliance with referrals (44, 45). Disrespectful maternal health care is also a sign of a fractured health system in terms of quality and accountability and it expresses power dynamics that conspire against both patients and providers (45, 46). Disrespectful and abusive behaviour from health care providers might reflect the poor working and living conditions that health workers experience in this rural settings: delays in disbursement of funds from the central government, shortage of health workers, unclear mechanisms for accountability, lack of incentives to motivate overburdened staffs and lack of guidelines for partnership development (47, 48).

## Limitations of the study

This study has a number of limitations. The use of FGDs was a challenge as some participants were shy and did not share much information. However, the researcher tried to enhance participation and reminded the participants that all issues that were discussed should remain within the group. The way the participants were selected, from health care facilities, might have led to social desirability and exclusion of women with the poorest experiences with health care services and/or those from the most disadvantaged backgrounds. We also did not consider the place where women gave birth, education, marital status, and type of employment when selecting the participants. It would have been interesting to explore whether variations in perceptions of women by some of these characteristics existed.

## Conclusions

This study shows that women in the district were aware that maternal health care services could be beneficial during pregnancy and delivery. However, awareness of postpartum complications and the role that medical services could play during that stage was poor. Multiple barriers to uptake of services were identified, such as the costs of transport; formal and informal payments; use of traditional birth attendants; and perceived low quality of services due lack of resources and disrespectful attitudes of providers. These barriers tend to have a cumulative effect where poverty remains the root cause. These findings call for improvement on the services provided. Improvements should address, accessibility of services, professionals' attitudes and stronger promotion of the importance of postpartum check-ups. Finally, the health system could also benefit from establishing ways to gather and assess users’ views on the services offered, and to channel their complaints to the appropriate authorities.

## References

[1] UNFPA (2005). Needs assessment of obstetric fistula in selected zones in Somalia: final report.

[2] TDHS (2010). Key findings.

[3] Ronsmans C, Graham WJ (2006). Maternal survival series steering group: maternal mortality: who, when, where, and why. Lancet.

[4] Lawn J, Kerber K (2006). Opportunity for Africa's newborns: practical data, policy and programmatic support for newborn care in Africa.

[5] Kinney MV, Kerber KJ, Black RE, Cohen B, Nkrumah F, Coovadia H (2010). Sub-Saharan Africa's mothers, newborns, and children: where and why do they die?. PLoS Med.

[6] Gil-González D, Carrasco-Portiño M, Ruiz MT (2006). Knowledge gaps in scientific literature on maternal mortality: a systematic review. Bull World Health Organ.

[7] Gabrysch S, Campbell OMR (2009). Still too far to walk: literature review of the determinants of delivery services use. BMC Pregnancy Childbirth.

[8] Hogan CM, Foreman KJ, Naghavi M, Aha SY, Wang M, Makela SM (2010). Maternal mortality for 181 countries 1980–2008: a systematic analysis of progress towards Millennium Development Goal 5. Lancet.

[9] UNHCR and WHO (2008). The right to health.

[10] UNFPA (2003). State of the world population: making one billion count.

[11] Hunt P, Bueno deMesquita J (2006). The rights to sexual and reproductive health.

[12] Braveman P, Gruskin S (2003). Poverty, equity, human rights and health. Bull World Health Organ.

[13] The United Republic of Tanzania (2011). Comprehensive council health plan for the year 2011/2012.

[14] The United Republic of Tanzania (2007). Tanzania health policy.

[15] NBS (2011). Tanzania demographic and health survey 2010.

[16] NBS (2007). Household budget survey 2007. Final Report, United Republic of Tanzania, President's Office, Planning and Privatization.

[17] Sorensen K, Van den Broucke S, Fullam J (2012). Health literacy and public health: a systematic review and integration of definitions and models. BMC Public Health.

[18] Mselle LT, Kohi TW, Mvungi A, Evjen-Olsen B, Moland KM (2011). Waiting for attention and care: birthing accounts of women in rural Tanzania who developed obstetric fistula as an outcome of labour. BMC Pregnancy Childbirth.

[19] Mpembeni NMR, Killewo ZJ, Leshabari TM, Masawe SN, Jahn A, Mushi D (2007). Use pattern of maternal health services and determinants of skilled care during delivery in Southern Tanzania: implications for achievements of MDG 5 targets. BMC Pregnancy Childbirth.

[20] Mbekenga CK, Lugina HI, Olsson P (2011). Postpartum experiences of first-time mothers in a Tanzanian suburb. Women Birth.

[21] Mrisho M, Obrist B, Schellenberg AM, Haws RA, Mushi AK, Mshinda H (2009). The use of antenatal and postnatal care: perspectives and experiences of women and health care providers in rural southern Tanzania. BMC Pregnancy Childbirth.

[22] Magoma M, Requejo J, Campbell OM, Cousens S, Filippi V (2010). High ANC coverage and low skilled attendance in a rural Tanzanian district: a case for implementing a birth plan intervention. BMC Pregnancy Childbirth.

[23] The United Republic of Tanzania (2009). Secondary statistical report.

[24] WHO, UNICEF, UNFPA, World Bank (2012). Maternal mortality in 1990 to 2010.

[25] Mahiti GR, Kiwara AD, Mbekenga CK, Hurtig AK, Goicolea I (2015). We have been working overnight without sleeping: traditional birth attendants’ practices and perceptions of post-partum care services in rural Tanzania. BMC Pregnancy Child Birth.

[26] Creswell JW (1998). Qualitative inquiry and research design among five traditions.

[27] Graneheim UH, Lundman B (2004). Qualitative content analysis in nursing research: concepts, procedures and measures to achieve trust-worthiness. Nurse Educ Today.

[28] Open code 3.7 (2007). http://www.umu.se/phmed/epidemi/forskning.open_code.html.

[29] Lincoln YS, Guba EG (1985). Naturalistic inquiry.

[30] Malabika S, Schimid G, Larsson E, Kirenga S, De Allegri M, Newhann F (2010). Quality of antenatal care in rural southern Tanzania: a reality check. BMC Res Notes.

[31] Mbekenga CK (2011). Striving to promote family health after childbirth. Studies in low-income suburbs of Dar es Salaam, Tanzania.

[32] Cook JR, Galli BMB (2004). Invoking human rights to reduce maternal deaths. Lancet.

[33] Gruskin S (2008). Reproductive and sexual rights: do words matter?. Am J Public Health.

[34] Shaw D (2006). Sexual and reproductive health: rights and responsibilities. Lancet.

[35] Mujanja SP, Tsitsi M, Kandawasvika G, Hussein J, Binns MC, Webber R (2012). Geographical access, transport and referral systems. Maternal and perinatal health in developing countries.

[36] Urassa E, Lindmark G, Nystrom L (1995). Maternal mortality in Dar es Salaam, Tanzania; Socio-economic, obstetric history and accessibility of health care factors. Afr J Health Sci.

[37] Kruk EM, Paczkowski M, Mbaruku G, de Pinho H, Galea S (2009). Women's preference for place of delivery in rural Tanzania: a population based discrete choice experiment. Am J Public Health.

[38] PHRplus (2013). Health Systems Assessment Approach: A HOW- TO Manual.

[39] Lwelamira J, Safari J (2012). Choice of place for child birth: prevalence and determinants of health facility delivery among women in Bahi district, Central Tanzania. Asian J Medical Sci.

[40] Mubyazi GM, Bloch P, Magnussen P, Olsen OE, Byskov J, Hansen KS (2010). Women's experiences and views about costs of seeking malaria chemoprevention and other antenatal services: a qualitative study from two districts in rural Tanzania. Malar J.

[41] Kowalenski M, Mujinja P, Jahn A (2002). Can mothers afford maternal health care costs? User costs of maternal services in rural Tanzania. Afr J Reprod Health.

[42] Ebuehi OM, Akintujole L (2012). Perception and utilization of traditional birth attendants by pregnant women attending primary health care clinics in a rural Local Government Area in Ogun state, Nigeria. Int J Womens Health.

[43] Jewkes R, Abrahams N, Mvo Z (1998). Why do nurses abuse patients? Reflections from South African obstetric services. Social Sci and Medicine.

[44] Pembe AB, Urassa DP, Darj E, Carlstedt A, Olsson P (2008). Qualitative study on maternal referrals in rural Tanzania: decision making and acceptance of referral advice. Afr J Reproductive Health.

[45] Kruk ME, Kujawski S, Mbaruku G, Ramsey K, Moyo W, Freedman LP (2014). Disrespective and abuse treatment during facility delivery in Tanzania: a facility and community survey. Health Policy Plann.

[46] Freedman LP, Kruk ME (2014). Disrespect and abuse of women in childbirth: challenging the global, quality and accountability agendas. Lancet.

[47] Mkoka DA, Kiwara A, Goicolea I, Hurtig AK (2014). Governing the implementation of emergence obstetric care: experiences of rural district health managers, Tanzania. BMC Health Serv Res.

[48] Mkoka DA, Mahiti GR, Kiwara A, Mwangu M, Goicolea I, Hurtig AK (2015). ‘Once the government employs you, it forgets you’. Health workers’ and managers perspectives on factors influencing working conditions for provision of maternal care services in a rural district of Tanzania. BMC Human Resources for Health.

